# Baseline imbalance and heterogeneity are present in meta-analyses of randomized clinical trials examining the effects of exercise and medicines for blood pressure management

**DOI:** 10.1038/s41440-022-00984-3

**Published:** 2022-07-26

**Authors:** Michael A. Wewege, Harrison J. Hansford, Brishna Shah, Yannick L. Gilanyi, Susan R. G. Douglas, Belinda J. Parmenter, James H. McAuley, Matthew D. Jones

**Affiliations:** 1 School of Health Sciences, Faculty of Medicine and Health, University of New South Wales, Sydney, NSW Australia; 2 Centre for Pain IMPACT, Neuroscience Research Australia, Sydney, NSW Australia

**Keywords:** Bias, Clinical Trial, Meta-Analysis, Systematic Review

## Abstract

Randomized clinical trials attempt to reduce bias and create similar groups at baseline to infer causal effects. In meta-analyses, baseline imbalance may threaten the validity of the treatment effects. This meta-epidemiological study examined baseline imbalance in comparisons of exercise and antihypertensive medicines. Baseline data for systolic blood pressure, diastolic blood pressure, and age were extracted from a network meta-analysis of 391 randomized trials comparing exercise types and antihypertensive medicines. Fixed-effect meta-analyses were used to determine the presence of baseline imbalance and/or inconsistency. Meta-regression analyses were conducted on sample size, the risk of bias for allocation concealment, and whether data for all randomized participants were presented at baseline. In one exercise comparison, the resistance group was 0.3 years younger than the control group (95% confidence interval 0.6 to 0.1). Substantial inconsistency was observed in other exercise comparisons. Less data were available for medicines, but there were no occurrences of baseline imbalance and only a few instances of inconsistency. Several moderator analyses identified significant associations. We identified baseline imbalance as well as substantial inconsistency in exercise comparisons. Researchers should consider conducting meta-analyses of key prognostic variables at baseline to ensure balance across trials.

## Graphical abstract

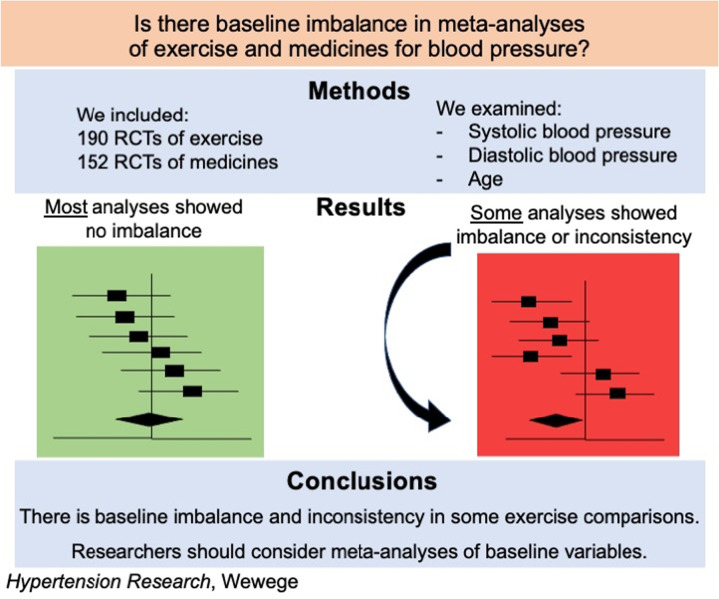

## Introduction

A meta-analysis of randomized clinical trials (RCTs) synthesizes evidence to inform clinical guidelines and policies [1, 2]. RCTs attempt to reduce bias and establish two or more similar groups at baseline, suggesting that any subsequent differences between groups are caused by allocated interventions. An imbalance in prognostic factors (i.e., age or condition severity) between groups may occur at baseline within a trial by chance [1, 3, 4]. There are also nonrandom reasons. Insecure allocation concealment (investigators influencing the allocation of participants to groups) may bias the results [2, 5, 6]. Deviating from the intention-to-treat analysis method (such as a “per-protocol” analysis) and reporting data for only a subset of the randomized participants may also distort the results of RCTs [7, 8].

Meta-analysis of baseline values may indicate evidence of bias at baseline for important prognostic factors, which may threaten the validity of the results [1–3, 9]. In a meta-analysis of calcium supplements for weight loss, the authors found that participants allocated to the treatment groups had a body mass that was 1.73 kg (95% confidence interval [CI] 2.97 to 0.5) lower than that of participants allocated to the control groups [1]. When this baseline difference was statistically controlled in an updated meta-analysis and meta-regression, the effect of calcium supplements was no longer statistically significant, suggesting that the result from the original meta-analysis was largely due to the baseline imbalance in body mass [1]. Similarly, in a meta-analysis of oseltamivir for treating flu, the author found that fewer participants who tested positive for influenza were assigned to the treatment group than to the control group (relative risk = 0.95, 95% CI 0.91 to 0.99), suggesting bias in treatment allocation [9]. Baseline imbalances have also been identified in other studies [2, 3, 10].

Recently, we observed baseline imbalance in our meta-analysis examining the antihypertensive effects of isometric exercise compared to nonexercise controls in adults with elevated blood pressure [11]. The pooled baseline systolic blood pressure (SBP) was 4.78 mmHg higher (95% CI 4.03 to 5.52) in the exercise group than the control group, and the pooled baseline diastolic blood pressure (DBP) was 5.48 mmHg higher (95% CI 5.10 to 5.68) in the exercise group than the control group. This may indicate bias across the studies and question the validity of the estimated treatment effects, given that the pooled exercise group was, on average, substantially different from the control group in the outcome of interest at baseline. These differences were largely driven by one comparatively large study (*n* = 400) with a large baseline imbalance between the intervention and control groups [12]. When we removed this one study, the baseline imbalance and heterogeneity were largely attenuated (SBP 0.64 mmHg, 95% CI −0.58 to 1.85; DBP 1.23 mmHg, 95% CI 0.13 to 2.34).

Therefore, this meta-epidemiological study examined baseline imbalance in the comparisons of various exercise and antihypertensive medicines. It also examined whether sample size and/or the risk of selection bias were associated with potential baseline imbalances.

## Methods

We preregistered the protocol for this study on the Open Science Framework (osf.io/dgu9b). We obtained the datasets of 391 RCTs (197 exercise trials and 194 antihypertensive medicine trials) used in a network meta-analysis of different modes of exercise and classes of antihypertensive medicines that was published in a leading sports medicine journal and has had substantial impact in its field [13].

One author (MAW) identified the manuscripts of each included RCT. Two authors (MAW and one of HJH, BS, YLG, or SRGD) independently extracted the number of participants and mean and standard deviation (SD) values at baseline for three outcomes: SBP, DBP, and age. We only extracted data for the groups included in the network meta-analysis. We preferentially extracted baseline data from all randomized participants in each RCT (typically outlined in Table 1 of a study), followed by data for participants who were analyzed. Discrepancies between authors were resolved via discussion and arbitration with a third author (MDJ) if needed. Where necessary, we transformed values reported in other forms to the mean and SD [14]; these data were most commonly presented as the median and range/interquartile range. We extracted data from figures using *WebPlotDigistizer* [15].

**Table 1:** Results for systolic blood pressure at baseline from 190 exercise studies

	No. studies (participants)		Fixed-effect meta-analysis		Moderator analyses		
Comparison	Total	Data available	Mean difference (95% CI)	Heterogeneity	Sample size	Allocation concealment	Data from all participants
Endurance vs. control	131 (6754)	128 (6539)	0.0 (−0.5, 0.5)	Q = 148.4, *p* = 0.095, *I*^*2*^ = 14.4%	0.00 (0.00, 0.00), *p* = 0.87	0.23 (−1.58, 2.05), *p* = 0.80	−0.14 (−1.15, 0.86), *p* = 0.78
Resistance vs. control	45 (1481)	45 (1481)	0.0 (−1.2, 1.1)	Q = 65.7, *p* = 0.02, *I*^*2*^ = 33.0%	−0.02 (−0.09, 0.06), *p* = 0.63	−1.50 (−7.17, 4.17), *p* = 0.60	1.04 (−1.26, 3.35), *p* = 0.37
Isometric vs. control	12 (376)	11 (356)	−0.2 (−1.9, 1.5)	Q = 6.7, *p* = 0.75, *I*^*2*^ = 0.0%	0.01 (−0.19, 0.22), *p* = 0.90	NA^a^	−0.26 (−4.38, 3.86), *p* = 0.90
Combined vs. control	25 (1254)	25 (1254)	−0.2 (−1.4, 1.0)	Q = 16.8, *p* = 0.86, *I*^*2*^ = 0.0%	0.00 (−0.01, 0.01), *p* = 0.95	−2.26 (−7.96, 3.44), *p* = 0.44	0.92 (−1.55, 3.40), *p* = 0.46
Endurance vs. resistance	17 (533)	16 (515)	0.0 (−2.1, 2.2)	Q = 8.8, *p* = 0.89, *I*^*2*^ = 0.0%	−0.05 (−0.17, 0.07), *p* = 0.42	1.54 (−6.42, 9.50), *p* = 0.70	−1.51 (−6.76, 3.74), *p* = 0.57
Endurance vs. combined	17 (536)	16 (496)	−0.2 (−1.8, 1.4)	Q = 10.5, *p* = 0.78, *I*^*2*^ = 0.0%	0.10 (−0.06, 0.26), *p* = 0.21	−0.55 (−2.36, 1.25), *p* = 0.55	0.21 (−3.03, 3.46), *p* = 0.90
Resistance vs. combined	9 (235)	9 (235)	−1.4 (−4.4, 1.5)	Q = 10.3, *p* = 0.25, *I*^*2*^ = 22.0%	0.13 (−0.51, 0.78), *p* = 0.69	4.77 (−7.68, 17.22), *p* = 0.45	7.79 (0.00, 15.57), *p* = 0.05
Endurance vs. isometric	Not performed because only three studies were available for this analysis						
Isometric vs. combined	Not performed because only one study was available for this analysis						

*CI* confidence interval, *NA* not available

^a^Moderator analysis not performed because all studies were in the same subgroup

Because our study focused on the level of exercise mode (e.g., endurance, resistance, isometric or combined exercise) or medicine type, we combined intervention groups within RCTs that used the same exercise mode or medicine type (e.g., groups that examined high-intensity and low-intensity endurance exercise or different dosages of the same medicine) [14].

Two authors (MAW and one of HJH, BS, YLG, or SRGD) independently appraised each study for risk of bias in allocation concealment using guidance from the Cochrane Risk of bias tool [16]. We noted studies where data were not available for all participants at baseline (such as studies that only presented baseline data for a per-protocol analysis). Discrepancies between authors were resolved via discussion and arbitration with a third author (MDJ). We did not contact the authors of the studies to request any data due to the large quantity of articles included in this study, as well as the substantial difference in publication dates among articles investigating exercise (mean = 2008, range 1976 to 2018) and medicines (mean = 1994, range 1968 to 2009). The mean difference in publication dates was 14 years (95% CI 13 to 17). Given the age of the medicine articles, many had no email contact available.

We noted four studies in the endurance vs. control comparison that provided duplicate baseline data. We removed the duplicate studies, leaving only the baseline data from the original study publications.

### Statistical analysis

We examined baseline imbalance for the outcomes of SBP, DBP, and age. We selected SBP and DBP because we observed baseline imbalance in these variables in our previous review, and they are often the primary outcomes in the management of hypertension [11]. We also selected age because it is a commonly reported variable, and an imbalance in this variable has been noted in previous research from other fields [2, 17]. Given that SBP, DBP, and age are related to other indicators of cardiometabolic health (e.g., body mass index), our outcome choices were likely to capture potential imbalances in other outcomes [18, 19]. The reduced number of outcomes also reduced our risk of type 1 error.

We examined baseline imbalance in the following comparisons:

- Endurance exercise vs. control groups.
- Resistance exercise vs. control groups.
- Isometric exercise vs. control groups.
- Combination exercise vs. control groups.
- Diuretic vs. control groups.
- Calcium channel blocker (CCB) vs. control groups.
- Beta-blocker vs. control groups.
- Angiotensin II receptor blocker (ARB) vs. control groups.
- Angiotensin-converting enzyme (ACE) inhibitor vs. control groups.

Data permitting, we also analyzed the following comparisons between two interventions:

- Endurance exercise vs. resistance exercise.
- Endurance exercise vs. isometric exercise.
- Endurance exercise vs. combination exercise.
- Resistance exercise vs. combination exercise.
- Isometric exercise vs. combination exercise.
- Diuretics vs. ACE inhibitors.
- Diuretics vs. ARBs.
- ACE inhibitors vs. ARBs.

We used a fixed-effect meta-analysis to compare groups, which is the appropriate model in this circumstance because the true effect is zero difference between groups [1–3, 17, 20]. We considered groups to be different at baseline if the mean difference and 95% CI did not cross zero. We quantified heterogeneity with the Cochran Q test and considered *I*^*2*^ > 30% to indicate substantial inconsistency [2, 17]. We conducted univariate meta-regression to examine the potential moderators of total sample size (continuous variable), risk of selection bias (Low risk or Unclear/High risk), and whether baseline data were available for all randomized participants (Yes or Unclear/No). We did not investigate the impact of moderators on absolute imbalance (which ignores the direction of the imbalance), as this may disguise random variation that is expected in baseline data and artificially induce false imbalances.

We performed a preplanned sensitivity analysis on the exercise trials that examined participants with a mean baseline SBP ≥ 140 mmHg (the definition of hypertension in the network meta-analysis). This was not required for antihypertensive medicines because all participants had hypertension.

## Results

### Exercise

Of 193 RCTs of exercise, 190 were included. All studies used a parallel group design, but three RCTs compared two doses of the same exercise type and therefore were not analyzed. We extracted data from figures for six studies. Seven studies had a low risk of bias for allocation concealment, four studies had a high risk of bias, and the remaining 179 studies had an unclear risk of bias. Ninety-four studies reported baseline data for all randomized participants.

#### All participants

There were no baseline imbalances in any comparisons for SBP (Table 1). There was substantial inconsistency in the resistance exercise vs. control groups comparison (*I*^*2*^ = 33.0%; Supplementary Fig. 1), as well as some evidence of inconsistency in the endurance exercise vs. control groups comparison (*I*^*2*^ = 14.4%) and in the resistance exercise vs. combined exercise comparison (*I*^*2*^ = 22.0%). No moderator analyses were statistically significant.

There were no baseline imbalances in any comparisons for DBP (Table 2). There was substantial inconsistency in the endurance exercise vs. control groups (*I*^*2*^ = 30.3%; Supplementary Fig. 2), resistance exercise vs. control groups (*I*^*2*^ = 41.0%; Supplementary Fig. 3), and resistance exercise vs. combined exercise (*I*^*2*^ = 35.4%: Supplementary Fig. 4) comparisons. Sample size was a significant moderator in the endurance exercise vs. control groups comparison (ß = 0.00 (95% CI 0.00 to 0.01), *p* < 0.01; increasing sample size associated with higher DBP in endurance exercise) and the resistance exercise vs. control groups comparison (ß = −0.06 (95% CI −0.11 to −0.01), *p* = 0.01; increasing sample size associated with higher DBP in control groups). Data from all participants at baseline were a significant moderator in the endurance exercise vs. control groups comparison (studies not reporting data for all participants at baseline had a significantly higher DBP in endurance exercise compared to studies reporting all data; difference between subgroups = 1.30 mmHg (95% CI 1.84 to 0.77), *p* < 0.01) and the resistance exercise vs. combined exercise comparison (studies not reporting data for all participants at baseline had a significantly higher DBP in the combined group compared to studies reporting all data; difference between subgroups = 5.15 mmHg (95% CI 0.23 to 10.08), *p* = 0.04).

**Table 2:** Results for diastolic blood pressure at baseline from 190 exercise studies

	No. studies (participants)		Fixed-effect meta-analysis		Moderator analyses		
Comparison	Total	Data available	Mean difference (95% CI)	Heterogeneity	Sample size	Allocation concealment	Data from all participants
Endurance vs. control	131 (6754)	123 (6394)	−0.3 (−0.5, 0.0)	Q = 175.0, *p* = 0.0012, *I*^*2*^ = 30.3%	0.00 (0.00, 0.01), *p* < 0.01	−0.61 (−1.76, 0.54), *p* = 0.30	−1.30 (−1.84, −0.77), *p* < 0.01
Resistance vs. control	45 (1481)	45 (1481)	0.1 (−0.7, 0.9)	Q = 74.6, *p* = 0.0027, *I*^*2*^ = 41.0%	−0.06 (−0.11, −0.01), *p* = 0.01	−1.18 (−4.89, 2.54), *p* = 0.54	1.29 (−0.37, 2.94), *p* = 0.13
Isometric vs. control	12 (376)	11 (356)	0.5 (−0.8, 1.8)	Q = 11.7, *p* = 0.31, *I*^*2*^ = 14.1%	−0.06 (−0.04, 0.21), *p* = 0.2	NA^a^	−0.43 (−3.27, 2.41), *p* = 0.77
Combined vs. control	25 (1254)	25 (1254)	−0.5 (−1.3. 0.3)	Q = 16.4, *p* = 0.87, *I*^*2*^ = 0.0%	0.0 (−0.01, 0.0), *p* = 0.33	−0.26 (−3.30, 2.78), *p* = 0.87	−0.63 (−2.20, 0.95), *p* = 0.44
Endurance vs. resistance	17 (533)	16 (515)	0.0 (−1.3, 1.3)	Q = 8.8, *p* = 0.89, *I*^*2*^ = 0.0%	0.04 (−0.04, 0.11), *p* = 0.32	0.09 (−4.77, 4.96), *p* = 0.97	−1.27 (−4.21, 1.68), *p* = 0.40
Endurance vs. combined	17 (536)	16 (496)	−0.2 (−1.3, 0.9)	Q = 11.3, *p* = 0.73, *I*^*2*^ = 0.0%	−0.04 (−0.15, 0.07), *p* = 0.51	−0.54 (−1.76, 0.67), *p* = 0.38	1.39 (−0.81, 3.58), *p* = 0.22
Resistance vs. combined	9 (235)	9 (235)	−0.1 (−2.1, 1.9)	Q = 12.4, *p* = 0.14, *I*^*2*^ = 35.4%	−0.02 (−0.41, 0.36), *p* = 0.90	−1.91 (−9.98, 6.16), *p* = 0.64	5.15 (0.23, 10.08), *p* = 0.04
Endurance vs. isometric	Not performed because only three studies were available for this analysis						
Isometric vs. combined	Not performed because only one study was available for this analysis						

*CI* confidence interval, *NA* not available

^a^Moderator analysis not performed because all studies were in the same subgroup

In the analysis of age (Table 3), there was baseline imbalance in the resistance vs. control groups comparison: the resistance group was 0.3 years younger (95% CI 0.6 to 0.1) than the control group. Inconsistency was detected in the endurance exercise vs. control groups (*I*^*2*^ = 14.1%), resistance exercise vs. control groups (*I*^*2*^ = 14.6%), combined exercise vs. control groups (*I*^*2*^ = 16.8%), and endurance exercise vs. resistance exercise (*I*^*2*^ = 13.4%) comparisons. Data from all participants at baseline were a significant moderator in the combined vs. control groups comparison (studies not reporting data for all participants at baseline had a significantly higher age in combined exercise compared to studies reporting all data; difference between subgroups = 1.20 years (95% CI 2.28 to 0.11), *p* = 0.03).

**Table 3:** Results for age at baseline from 190 exercise studies

	No. studies (participants)		Fixed-effect meta-analysis		Moderator analyses		
Comparison	Total	Data available	Mean difference (95% CI)	Heterogeneity	Sample size	Allocation concealment	Data from all participants
Endurance vs. control	131 (6754)	106 (5146)	0.0 (−0.3, 0.3)	Q = 122.3, *p* = 0.12, *I*^*2*^ = 14.1%	0.00 (0.00, 0.00), *p* = 0.54	0.37 (−0.71, 1.44), *p* = 0.50	0.42 (−0.97, 0.14), *p* = 0.14
Resistance vs. control	45 (1481)	41 (1278)	−0.3 (−0.6, −0.1)	Q = 46.8, *p* = 0.21, *I*^*2*^ = 14.6%	−0.04 (−0.08, −0.00), *p* = 0.06	1.05 (−1.92, 4.02), *p* = 0.49	−0.64 (−1.31, 0.04), *p* = 0.07
Isometric vs. control	12 (376)	10 (328)	−0.4 (−1.5, 0.7)	Q = 7.0, *p* = 0.64, *I*^*2*^ = 0.0%	−0.04 (−0.15, −0.08), *p* = 0.55	NA^a^	−0.59 (−4.33, 3.15), *p* = 0.76
Combined vs. control	25 (1254)	24 (1221)	0.0 (−0.6, 0.5)	Q = 27.7, *p* = 0.23, *I*^*2*^ = 16.8%	0.00 (−0.01, 0.00), *p* = 0.64	−0.87 (−3.30, 1.56), *p* = 0.48	−1.20 (−2.28, −0.11), *p* = 0.03
Endurance vs. resistance	17 (533)	16 (496)	0.1 (−0.7, 0.8)	Q = 17.3, *p* = 0.30, *I*^*2*^ = 13.4%	−0.06 (−0.13, 0.01), *p* = 0.08	1.02 (−3.49, 5.53), *p* = 0.66	0.65 (−0.96, 2.26), *p* = 0.43
Endurance vs. combined	17 (536)	15 (447)	0.6 (−0.2, 1.3)	Q = 10.6, *p* = 0.72, *I*^*2*^ = 0.0%	0.03 (−0.09, 0.16), *p* = 0.58	−0.15 (−1.50, 1.20), *p* = 0.83	0.76 (−0.86, 2.37), *p* = 0.36
Resistance vs. combined	9 (235)	9 (235)	−0.4 (−1.6, 0.7)	Q = 7.1, *p* = 0.53, *I*^*2*^ = 0.0%	0.08 (−0.15, 0.31), *p* = 0.50	1.59 (−6.36, 9.54), *p* = 0.69	−0.68 (−3.75, 2.39, *p* = 0.66
Endurance vs. isometric	Not performed because only three studies were available for this analysis						
Isometric vs. combined	Not performed because only one study was available for this analysis						

*CI* confidence interval, *NA* not available

^a^Moderator analysis not performed because all studies were in the same subgroup

#### Participants with hypertension only

There were no baseline imbalances in any comparisons for SBP (Supplementary Table 1). There was substantial inconsistency in the endurance exercise vs. control groups comparison (*I*^*2*^ = 44.9%; Supplementary Fig. 5). Sample size was a significant moderator in the endurance exercise vs. control groups comparison (ß = 0.02 (95% CI 0.01 to 0.03), *p* < 0.01; increasing sample size was associated with higher SBP in endurance exercise).

There were no baseline imbalances or any inconsistencies in any comparisons for DBP (Supplementary Table 2). Sample size was a significant moderator in the endurance exercise vs. control groups comparison (ß = 0.01 (95% CI 0.00, 0.01), *p* < 0.01; increasing sample size was associated with higher DBP in endurance exercise).

There were no baseline imbalances in any comparisons for age (Supplementary Table 3). There was substantial inconsistency in the combined exercise vs. control groups comparison (*I*^*2*^ = 34.8%; Supplementary Fig. 6). Data from all participants at baseline were also a significant moderator in the combined vs. control groups comparison (studies not reporting data for all participants at baseline had a significantly higher age in combined exercise compared to studies reporting all data; difference between subgroups = 3.09 years (95% CI 5.81 to 0.36), *p* = 0.03).

### Medicines

Of 194 RCTs of antihypertensive medicines, 152 were included. We were unable to analyze 42 crossover RCTs because the data were not presented separately for groups at baseline (39 compared beta-blockers to placebos and 3 compared diuretics to placebos). We extracted data from figures for four studies. One study had a low risk of bias for allocation concealment, and the remaining 151 studies had an unclear risk of bias. Baseline data were reported for all randomized participants in 105 studies, with the remaining studies either not reporting data for all participants (*n* = 34) or having insufficient information to determine a judgment (*n* = 13).

None of the comparisons in SBP (Table 4), DBP (Table 5), or age (Table 6) displayed evidence of baseline imbalance. There was inconsistency for SBP in the ACE inhibitor vs. control groups comparison (*I*^*2*^ = 16.0%) and in the CCB vs. control groups comparison (*I*^*2*^ = 5.8%). There was inconsistency for DBP in the beta-blocker vs. control groups (*I*^*2*^ = 3.6%), ARB vs. control groups (*I*^*2*^ = 16.7%), and CCB vs. control groups (*I*^*2*^ = 20.4%) comparisons. There was inconsistency for age in the diuretic vs. control groups (*I*^*2*^ = 5.8%), beta-blocker vs. control groups (*I*^*2*^ = 19.4%), and ACE inhibitor vs. control groups (*I*^*2*^ = 20.4%) comparisons. One moderator was significant: increasing sample size was associated with a higher baseline SBP in the beta-blocker group in the beta-blocker vs. control groups comparison (ß = 0.01 (95% CI 0.00 to 0.01), *p* < 0.01).

**Table 4:** Results for systolic blood pressure at baseline from 152 antihypertensive medicines studies

	No. studies (participants)		Fixed-effect meta-analysis		Moderator analyses		
Comparison	Total	Data available	Mean difference (95% CI)	Heterogeneity	Sample size	Allocation concealment	Data from all participants
Diuretic vs. control	46 (7181)	25 (4310)	−0.2 (−0.9, 0.5)	Q = 20.0, *p* = 0.70, *I*^*2*^ = 0.0%	0.00 (0.00, 0.00), *p* = 0.88	−0.21 (−2.80, 2.37), *p* = 0.87	−0.74 (−2.62, 1.14), *p* = 0.44
Beta-blocker vs. control	22 (3878)	15 (2363)	0.0 (−1.1, 1.2)	Q = 8.5, *p* = 0.86, *I*^*2*^ = 0.0%	0.01 (−0.01, 0.02), *p* = 0.33	NA^a^	−0.31 (−2.87, 2.25), *p* = 0.81
ACE inhibitor vs. control	57 (8160)	36 (5161)	0.1 (−0.7, 1.0)	Q = 41.7, *p* = 0.20, *I*^*2*^ = 16.0%	0.00 (0.00, 0.00), *p* = 0.93	NA^a^	−0.35 (−3.38, 2.68), *p* = 0.82
ARB vs. control	36 (10406)	23 (7425)	−0.1 (−0.8, 0.5)	Q = 19.4, *p* = 0.62, *I*^*2*^ = 0.0%	0.00 (0.00, 0.00), *p* = 0.60	NA^a^	−0.08 (−2.87, 2.71), *p* = 0.95
CCB vs. control	9 (1050)	5 (520)	−0.1 (−1.4, 1.3)	Q = 4.3, *p* = 0.38, *I*^*2*^ = 5.8%	0.02 (−0.02, 0.05), *p* = 0.34	NA^a^	−1.23 (−6.29, 3.83), *p* = 0.63
Diuretic vs. ACE inhibitor	7 (698)	3 (206)	Not performed because only three studies provided data for this analysis				
Diuretic vs. ARB	Not performed because only five studies were available for this analysis						
ACE inhibitor vs. ARB	Not performed because only six studies were available for this analysis						

*ACE* angiotensin-converting enzyme, *ARB* angiotensin II receptor blocker, *CCB* calcium channel blocker, *CI* confidence interval, *NA* not available

^a^Moderator analysis not performed because all studies were in the same subgroup

**Table 5:** Results for diastolic blood pressure at baseline from 152 antihypertensive medicines studies

	No. studies (participants)		Fixed-effect meta-analysis		Moderator analyses		
Comparison	Total	Data available	Mean difference (95% CI)	Heterogeneity	Sample size	Allocation concealment	Data from all participants
Diuretic vs. control	46 (7181)	25 (4403)	0.1 (−0.2, 0.3)	Q = 15.0, *p* = 0.94, *I*^*2*^ = 0.0%	0.00 (0.00, 0.00), *p* = 0.85	0.06 (−0.79, 0.91), *p* = 0.89	−0.06 (−0.65, 0.54), *p* = 0.85
Beta-blocker vs. control	22 (3878)	18 (3287)	0.0 (−0.4, 0.3)	Q = 17.6, *p* = 0.41, *I*^*2*^ = 3.6%	0.00 (0.00, 0.01), *p* = 0.25	NA^a^	−0.44 (−1.25, 0.37), *p* = 0.29
ACE inhibitor vs. control	57 (8160)	40 (6040)	0.2 (−0.1, 0.4)	Q = 31.8, *p* = 0.79, *I*^*2*^ = 0.0%	0.00 (0.00, 0.00), *p* = 0.42	NA^a^	−0.38 (−1.18, 0.43), *p* = 0.36
ARB vs. control	36 (10406)	23 (7425)	0.1 (−0.2, 0.3)	Q = 26.4, *p* = 0.23, *I*^*2*^ = 16.7%	0.00 (0.00, 0.00), *p* = 0.26	NA^a^	−0.44 (−1.50, 0.62), *p* = 0.41
CCB vs. control	9 (1050)	5 (520)	−0.1 (−0.9, 0.6)	Q = 5.0, *p* = 0.28, *I*^*2*^ = 20.4%	−0.01 (−0.02, 0.01), *p* = 0.45	NA^a^	−1.25 (−3.24, 0.74), *p* = 0.22
Diuretic vs. ACE inhibitor	7 (698)	3 (206)	Not performed because only three studies provided data for this analysis				
Diuretic vs. ARB	Not performed because only five studies were available for this analysis						
ACE inhibitor vs. ARB	Not performed because only six studies were available for this analysis						

*ACE* angiotensin-converting enzyme, *ARB* angiotensin II receptor blocker, *CCB* calcium channel blocker, *CI* confidence interval, *NA* not available

^a^Moderator analysis not performed because all studies were in the same subgroup

**Table 6:** Results for age at baseline from 152 antihypertensive medicines studies

	No. studies (participants)		Fixed-effect meta-analysis		Moderator analyses		
Comparison	Total	Data available	Mean difference (95% CI)	Heterogeneity	Sample size	Allocation concealment	Data from all participants
Diuretic vs. control	46 (7181)	24 (4458)	−0.3 (−0.9, 0.2)	Q = 24.4, *p* = 0.38, *I*^*2*^ = 5.8%	0.00 (0.00, 0.00), *p* = 0.74	−1.06 (−3.64, 1.52), *p* = 0.42	−0.67 (−2.29, 0.96), *p* = 0.42
Beta-blocker vs. control	22 (3878)	14 (2828)	−0.1 (−0.9, 0.6)	Q = 16.1, *p* = 0.24, *I*^*2*^ = 19.4%	0.01 (0.00, 0.01), *p* < 0.01	NA^a^	1.84 (0.14, 3.54), *p* = 0.03
ACE inhibitor vs. control	57 (8160)	43 (6186)	−0.4 (−0.9, 0.1)	Q = 51.5, *p* = 0.15, *I*^*2*^ = 18.5%	0.00 (0.00, 0.01), *p* = 0.05	NA^a^	0.33 (−1.20, 1.68), *p* = 0.67
ARB vs. control	36 (10406)	27 (8285)	−0.4 (−0.8, 0.1)	Q = 21.7, *p* = 0.70, *I*^*2*^ = 0.0%	0.00 (0.00, 0.00), *p* = 0.48	NA^a^	0.36 (−0.61, 1.32), *p* = 0.47
CCB vs. control	9 (1050)	6 (595)	1.2 (−0.3, 2.6)	Q = 2.5, *p* = 0.78, *I*^*2*^ = 0.0%	−0.02 (−0.06, 0.01), *p* = 0.22	NA^a^	−0.95 (−5.43, 3.52), *p* = 0.68
Diuretic vs. ACE inhibitor	7 (698)	2 (186)	Not performed because only two studies provided data for this analysis				
Diuretic vs. ARB	Not performed because only five studies were available for this analysis						
ACE inhibitor vs. ARB	Not performed because only six studies were available for this analysis						

*ACE* angiotensin-converting enzyme, *ARB* angiotensin II receptor blocker, *CCB* calcium channel blocker, *CI* confidence interval, *NA* not available

^a^Moderator analysis not performed because all studies were in the same subgroup

## Discussion

Our meta-epidemiological study found one occurrence of baseline imbalance in the exercise comparisons and several occurrences of substantial inconsistency. It is the first to use a network meta-analysis as the data source, allowing us to explore a comprehensive dataset that reflects a large body of literature across different guideline-recommended types of exercise and medicines for the management of hypertension.

We observed one instance of pooled baseline imbalance of 0.3 years in the resistance vs. control groups comparison. A statistically significant difference at baseline should not be present, assuming the individual trials included in the analysis are methodologically sound and reported accurately. This may indicate a failure in the methodological procedures of some studies. None of our moderator analyses found statistically significant associations with this imbalance, but these are limited by poor reporting. Previous research noted pooled baseline imbalance in age in several meta-analyses [2], arguing that it is a marker for poor allocation practices that weaken the strength of a review’s conclusions. There may be methodological bias within some types of exercise for the management of hypertension.

There were no baseline imbalances in SBP or DBP in the exercise comparisons, contrary to our previous research [11]. We did observe substantial inconsistency in several comparisons, which is consistent with previous research [2, 17, 20]. This may be due to a few outlying trials within an analysis that contain marked baseline imbalance. For example, the SBP of the resistance exercise group in the Conceicao et al. study was 26.6 mmHg higher than that of the control group at baseline [21]. This study was an outlier, which may contribute to the inconsistency because other studies in this comparison had SBPs that ranged from 10.6 mmHg *higher* in the resistance group to 11.0 mmHg *lower* in the resistance group. The minimal clinically important difference for SBP is approximately 5 mmHg [11], indicating that some of these baseline imbalances are several-fold greater than the minimal clinically important difference. Outlying values may indicate bias within a study (e.g., poor randomization or allocation procedures) [2]. The results will also differ markedly depending on whether the change score or follow-up score is used in a meta-analysis [22]. The Conceicao et al. study reported that the SBP of the resistance exercise group changed from 138.4 mmHg to 130.8 mmHg, while that of the control group increased from 111.8 mmHg to 113.3 mmHg; if follow-up scores were used, the mean difference (17.5 mmHg) favored the control group, but if change scores were used, the mean difference favored the resistance group (−9.1 mmHg). The presence of heterogeneity/inconsistency at baseline in a meta-analysis can weaken its findings, especially if no attempt is made to adjust for these impacts [2].

We did not identify any baseline imbalance in the studies of medicines, and inconsistency did not reach our prespecified threshold for concern, although it may not be ignorable. However, it is important to note that fewer studies (but not fewer participants) were available for these comparisons because a) many studies used a crossover design, which could not be included, and b) there were many more missing data in these comparisons due to the age of the studies.

We observed that sample size and data availability at baseline were associated with the magnitude of the baseline difference in several comparisons, although not always favoring the intervention group, which would be the favorable direction if there is presumed investigator bias. The statistically significant associations between sample size and baseline imbalance were very small across all outcomes (ß ranging from 0.0 to 0.06), suggesting that these findings are likely to be spurious relationships with little meaning, driven by random imbalance in a larger trial in the analysis. However, the identification of marked imbalances in larger trials may also indicate further investigation over potential concerns about research integrity. We did not observe significant associations with allocation concealment, contrary to previous research [2]. However, these findings are limited by the small number of studies in some moderator analyses and poor reporting. Most studies of types of exercise and medicines did not clearly report their procedures for allocation concealment, which continues to be a concern for RCTs [23]. Given the much more recent publication of the exercise studies, this issue must be resolved to reduce the risk of bias. Compared to 47 medicine studies (31%), ninety-six exercise studies (51%) did not report baseline data for all randomized participants, instead only reporting data for participants who completed the study. This reporting threatens the internal validity of the results because missing data due to participant adherence in an RCT may not simply be ‘missing at random.’ RCTs should report baseline characteristics for all randomized participants in a publication irrespective of whether they completed the study.

Researchers who conduct meta-analyses in the field of exercise should initially consider conducting meta-analyses of baseline values of key prognostic variables to identify imbalances or heterogeneity [20]. We selected SBP, DBP, and age in this study because these variables are effect modifiers and are likely to encompass other cardiometabolic variables (e.g., body mass index). In other fields, different outcomes may be more relevant. Several methods have been proposed to explore heterogeneity/inconsistency in a meta-analysis of baseline values and identify ‘suspect’ RCTs; however, there is currently no consensus on how best to combat heterogeneity once it is identified [1, 2, 17, 20, 24, 25]. Some researchers promote individual patient data meta-analyses [1, 20, 25], while others propose simpler methods to identify and remove the individual studies that contribute the largest amount of baseline heterogeneity [20]. However, these methods cannot solve the underlying issue that some RCTs may not be conducted properly or may even be fraudulent [26]. Increasing methodological quality and reducing the risk of bias would improve the evidence base supporting the use of exercise and medicines in the management of hypertension.

## Conclusion

We identified baseline imbalance and inconsistency in meta-analyses of exercise and antihypertensive medicines. These results may indicate evidence of bias in randomization/allocation procedures in these trials, particularly in studies examining exercise. Systematic reviewers should conduct meta-analyses on important baseline characteristics.

## Supplementary information

Supplementary Material

## Data Availability

The data and codes from this study are available on the *Open Science Framework* (osf.io/dgu9b).
