# Supplementary material for: Baseline imbalance and heterogeneity are present in meta-analyses of randomized clinical trials examining the effects of exercise and medicines for blood pressure management
Source: Hypertens Res. 2022 Jul 26;45(10):1643–52. doi: 10.1038/s41440-022-00984-3 (PMC9474297; doi:10.1038/s41440-022-00984-3)
Supplement: Supplementary file 1 — Supplementary Material [file 41440_2022_984_MOESM1_ESM.docx]

**Baseline imbalance and heterogeneity are present in meta-analyses of randomised clinical trials examining the effects of exercise and medicines for blood pressure management**

**Supplementary data**

Michael A Wewege, Harrison J Hansford, Brishna Shah, Yannick L Gilanyi, Susan RG Douglas, Belinda J Parmenter, James H McAuley, Matthew D Jones

**Corresponding Author**

Michael Wewege

[m.wewege@unsw.edu.au](mailto:m.wewege@unsw.edu.au)

**Table of contents**

| **Supplementary Table 1.** Results for systolic blood pressure at baseline for hypertensive participants (baseline systolic ≥140 mmHg) from exercise studies. | 2 |
| --- | --- |
| **Supplementary Table 2.** Results for diastolic blood pressure at baseline for hypertensive participants (baseline systolic ≥140 mmHg) from exercise studies. | 3 |
| **Supplementary Table 3.** Results for age at baseline for hypertensive participants (baseline systolic ≥140 mmHg) from exercise studies. | 4 |
| **Supplementary Figure 1.** Forest plot for systolic blood pressure for the resistance vs control comparison with all participants. | 5 |
| **Supplementary Figure 2.** Forest plot for diastolic blood pressure for the endurance vs control comparison with all participants. | 6 |
| **Supplementary Figure 3.** Forest plot for diastolic blood pressure for the resistance vs control comparison with all participants. | 7 |
| **Supplementary Figure 4.** Forest plot for diastolic blood pressure for the resistance vs combined comparison with all participants. | 8 |
| **Supplementary Figure 5.** Forest plot for systolic blood pressure for the endurance vs control comparison with hypertensive participants (baseline systolic ≥140 mmHg). | 9 |
| **Supplementary Figure 6.** Forest plot for age for the combined vs control comparison with hypertensive participants (baseline systolic ≥140 mmHg). | 10 |

**Supplementary Table 1.** Results for systolic blood pressure at baseline for hypertensive participants (baseline systolic ≥140 mmHg) from exercise.

|  | **No. studies (participants)** | | **Fixed-effect meta-analysis** | | **Moderator analyses** | | |
| --- | --- | --- | --- | --- | --- | --- | --- |
| **Comparison** | **Total** | **Data available** | **Mean difference (95% CI)** | **Heterogeneity** | **Sample size** | **Allocation concealment** | **Data from all participants** |
| Endurance vs control | 33 (1589) | 33 (1589) | 0.5 (-0.4, 1.5) | Q = 58.1, *p* = 0.003,  *I^2^* = 44.9% | 0.02 (0.01, 0.03),  *p* < 0.01 | -0.59 (-4.22, 3.03),  *p* = 0.75 | -1.28 (-3.27, 0.71),  *p* = 0.21 |
| Resistance vs control | 6 (201) | 6 (201) | 2.6 (-1.1, 6.3) | Q = 2.7, *p* = 0.74,  *I^2^* = 0.0% | 0.05 (-0.19, 0.28), *p* = 0.70 | 0.48 (-7.85, 8.82), *p* = 0.91 | -0.68 (-8.30, 6.94),  *p* = 0.68 |
| Combined vs control | 7 (316) | 7 (316) | 1.4 (-09, 3.8) | Q = 4.6, *p* = 0.60,  *I^2^* = 0.0% | -0.03 (-0.11, 0.06), *p* = 0.54 | -5.92 (-17.49, 5.65), *p* = 0.32 | -0.78 (-5.47, 3.91),  *p* = 0.74 |
| Isometric vs  control | Not performed because only two studies were available for this analysis | | | | | | |
| Endurance vs resistance | Not performed because only three studies were available for this analysis | | | | | | |
| Endurance vs isometric | Not performed because no studies were available for this analysis | | | | | | |
| Endurance vs combined | Not performed because only four studies were available for this analysis | | | | | | |
| Resistance vs combined | Not performed because only one study was available for this analysis | | | | | | |
| Isometric vs combined | Not performed because no studies were available for this analysis | | | | | | |

NA, not available

* Moderator analysis not performed because all studies were in the same subgroup.

**Supplementary Table 2.** Results for diastolic blood pressure at baseline for hypertensive participants (baseline systolic ≥140 mmHg) from exercise studies.

|  | **No. studies (participants)** | | **Fixed-effect meta-analysis** | | **Moderator analyses** | | |
| --- | --- | --- | --- | --- | --- | --- | --- |
| **Comparison** | **Total** | **Data available** | **Mean difference (95% CI)** | **Heterogeneity** | **Sample size** | **Allocation concealment** | **Data from all participants** |
| Endurance vs control | 33 (1589) | 33 (1589) | 0.5 (0.0, 1.0) | Q = 29.0, *p* = 0.62,  *I^2^* = 0.0% | 0.01 (0.00, 0.01),  *p* < 0.01 | 0.09 (-2.21, 2.39),  *p* = 0.94 | -0.92 (-2.01, 0.16),  *p* = 0.10 |
| Resistance vs control | 6 (201) | 6 (201) | 0.5 (-1.7, 2.7) | Q = 2.6, *p* = 0.77,  *I^2^* = 0.0% | -0.04 (-0.18, 0.11), *p* = 0.61 | -0.29 (-5.75, 5.17), *p* = 0.92 | -1.38 (-3.39, 6.14),  *p* = 0.57 |
| Combined vs control | 7 (316) | 7 (316) | -1.4 (-2.8, 0.1) | Q = 2.2, *p* = 0.90,  *I^2^* = 0.0% | 0.00 (-0.06, 0.06), *p* = 0.94 | -0.28 (-6.53, 5.97), *p* = 0.93 | -1.11 (-4.03, 1.81),  *p* = 0.46 |
| Isometric vs  control | Not performed because only two studies were available for this analysis | | | | | | |
| Endurance vs resistance | Not performed because only three studies were available for this analysis | | | | | | |
| Endurance vs isometric | Not performed because no studies were available for this analysis | | | | | | |
| Endurance vs combined | Not performed because only four studies were available for this analysis | | | | | | |
| Resistance vs combined | Not performed because only one study was available for this analysis | | | | | | |
| Isometric vs combined | Not performed because no studies were available for this analysis | | | | | | |

NA, not available

* Moderator analysis not performed because all studies were in the same subgroup.

|  | **No. studies (participants)** | | **Fixed-effect meta-analysis** | | **Moderator analyses** | | |
| --- | --- | --- | --- | --- | --- | --- | --- |
| **Comparison** | **Total** | **Data available** | **Mean difference (95% CI)** | **Heterogeneity** | **Sample size** | **Allocation concealment** | **Data from all participants** |
| Endurance vs control | 33 (1589) | 28 (1415) | 0.2 (-0.3, 0.7) | Q = 22.1, *p* = 0.73,  *I^2^* = 0.0% | 0.00 (-0.01, 0.00),  *p* = 0.97 | 0.49 (-2.11, 3.09),  *p* = 0.71 | 0.11 (-1.02, 1.24),  *p* = 0.85 |
| Resistance vs control | 6 (201) | 5 (146) | -0.3 (-2.6, 2.0) | Q = 3.1, *p* = 0.54,  *I^2^* = 0.0% | 0.01 (-0.15, 0.18), *p* = 0.86 | 2.11 (-2.62, 6.85), *p* = 0.38 | -2.35 (-7.09, 2.39),  *p* = 0.33 |
| Combined vs control | 7 (316) | 6 (283) | 0.9 (-0.2, 2.0) | Q = 7.7, *p* = 0.18,  *I^2^* = 34.8% | 0.01 (-0.05, 0.08), *p* = 0.70 | -4.76 (-13.66, 4.13), *p* = 0.29 | -3.09 (-5.81, -0.36),  *p* = 0.03 |
| Isometric vs  control | Not performed because only two studies were available for this analysis | | | | | | |
| Endurance vs resistance | Not performed because only three studies were available for this analysis | | | | | | |
| Endurance vs isometric | Not performed because no studies were available for this analysis | | | | | | |
| Endurance vs combined | Not performed because only four studies were available for this analysis | | | | | | |
| Resistance vs combined | Not performed because only one study was available for this analysis | | | | | | |
| Isometric vs combined | Not performed because no studies were available for this analysis | | | | | | |

**Supplementary Table 3.** Results for age at baseline for hypertensive participants (baseline systolic ≥140 mmHg) from exercise studies.

NA, not available

* Moderator analysis not performed because all studies were in the same subgroup.


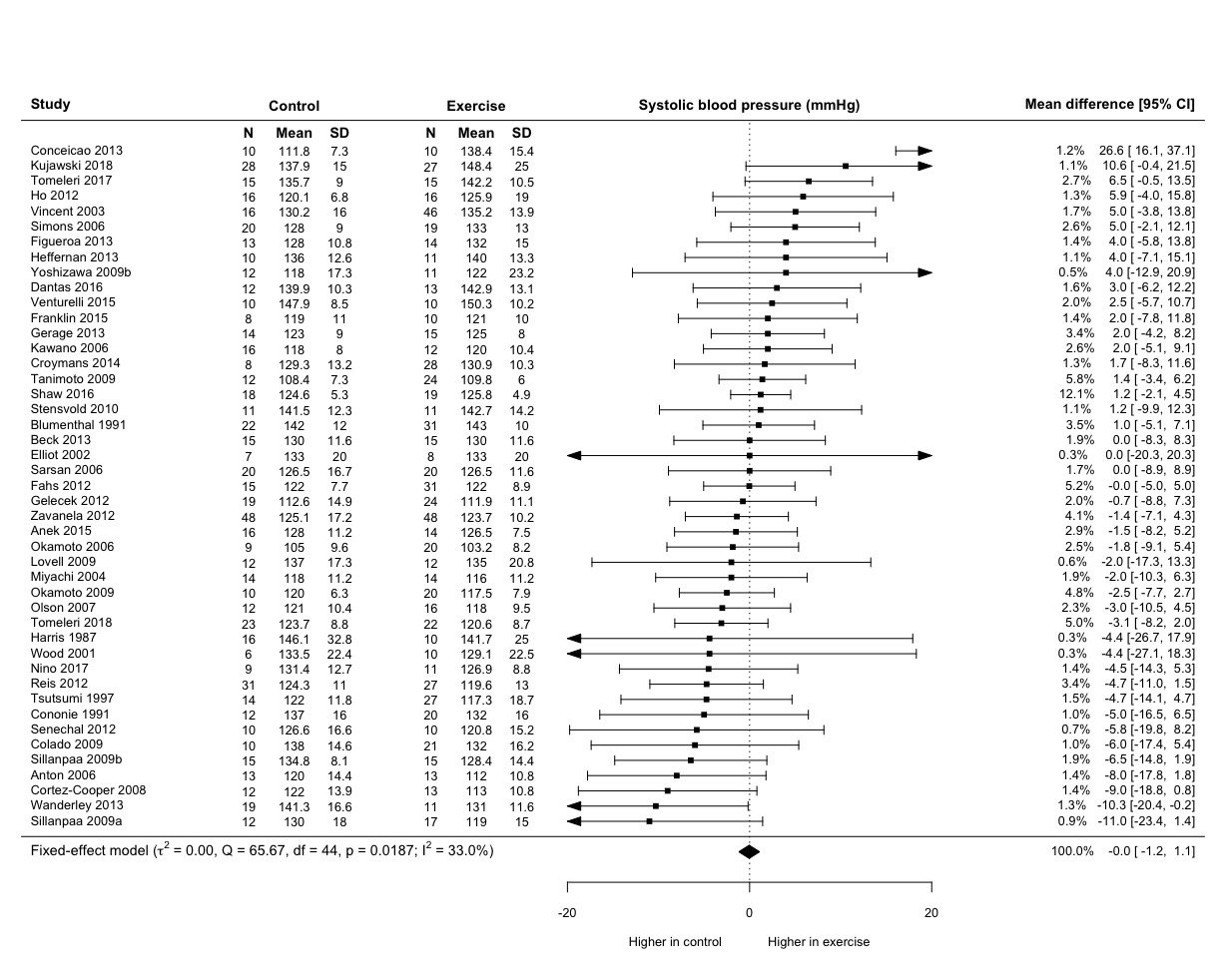


**Supplementary Figure 1.** Forest plot for systolic blood pressure for the resistance vs control comparison with all participants.


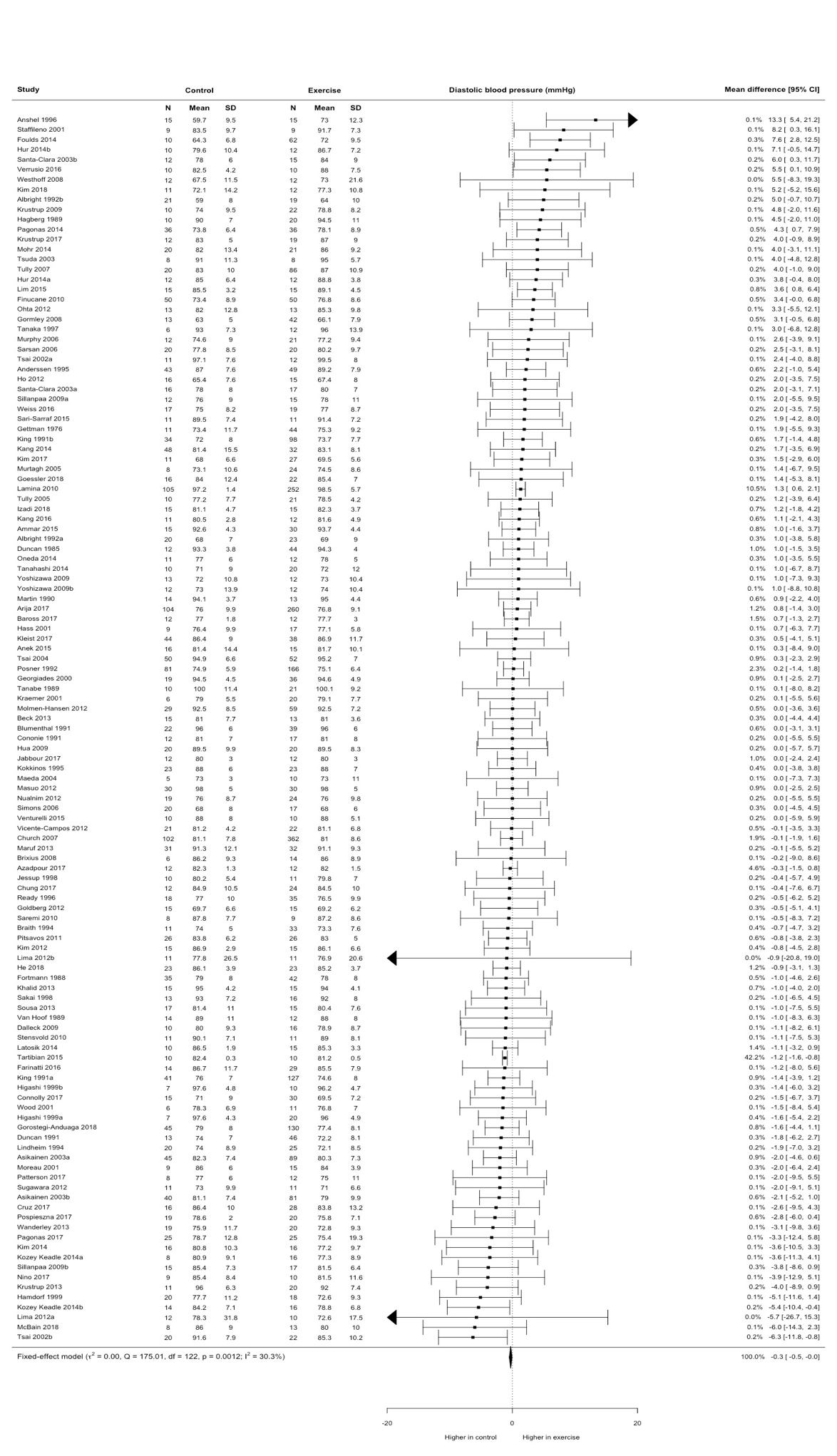


**Supplementary Figure 2.** Forest plot for diastolic blood pressure for the endurance vs control comparison with all participants.


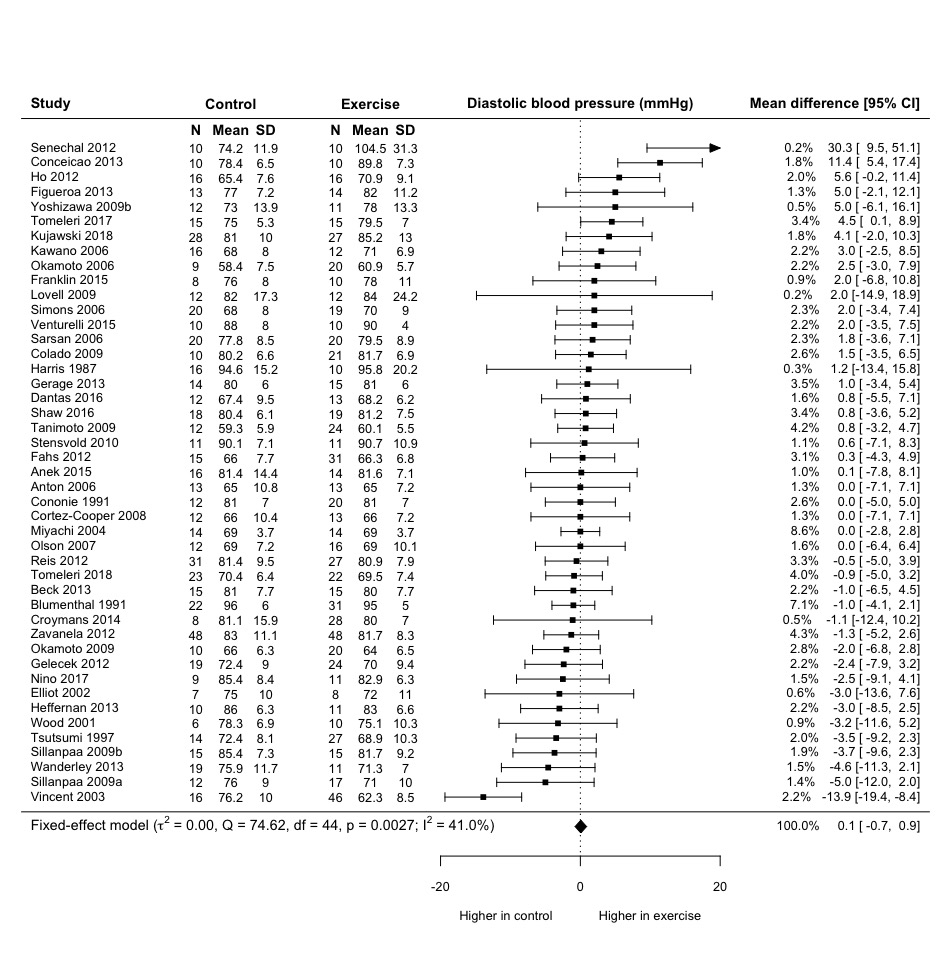


**Supplementary Figure 3.** Forest plot for diastolic blood pressure for the resistance vs control comparison with all participants.


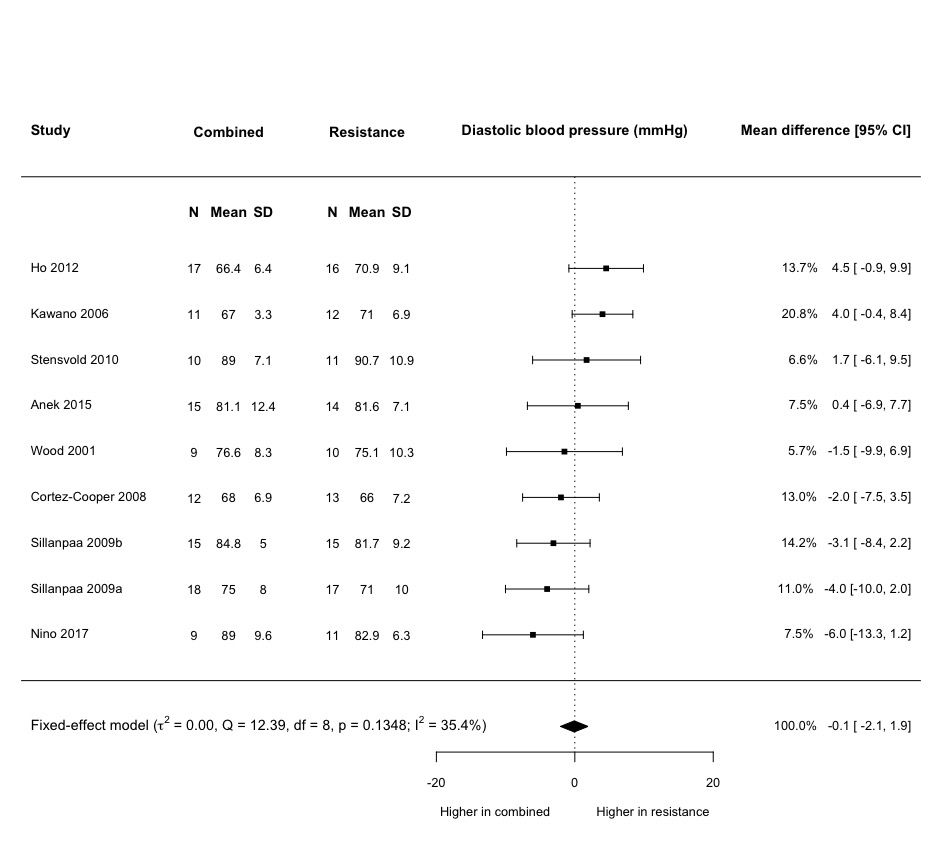


**Supplementary Figure 4.** Forest plot for diastolic blood pressure for the resistance vs combined comparison with all participants.


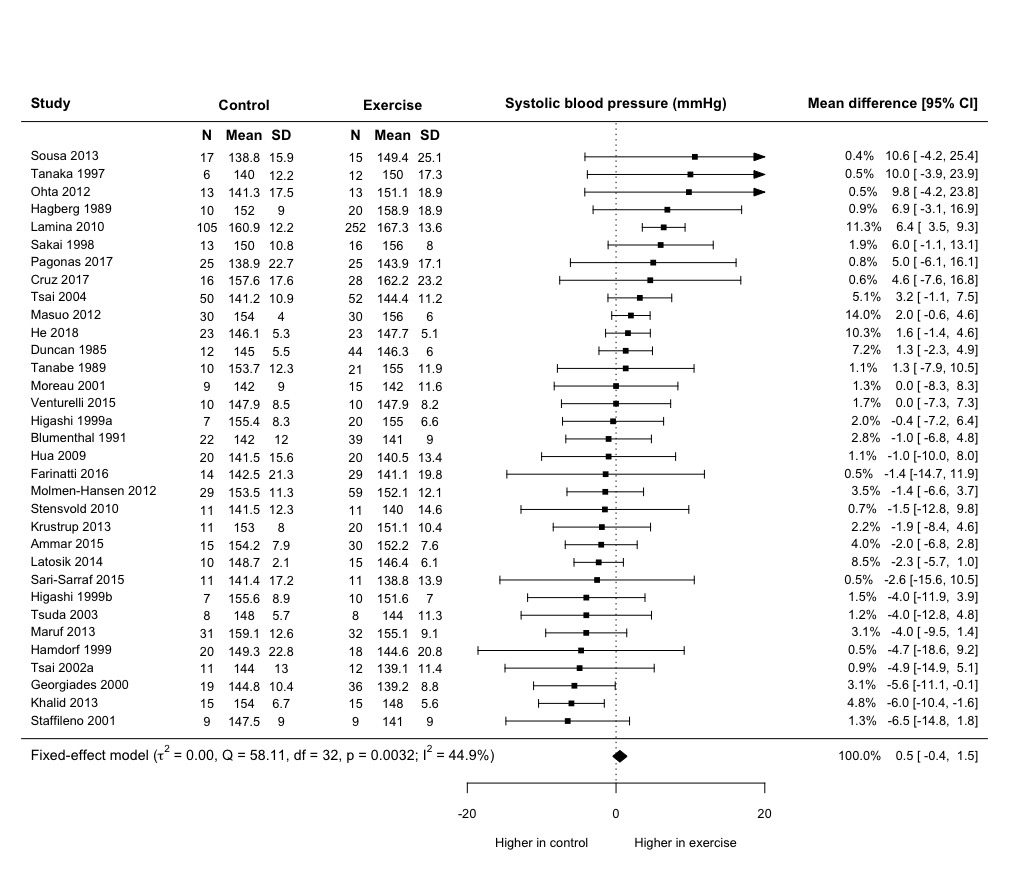


**Supplementary Figure 5.** Forest plot for systolic blood pressure for the endurance vs control comparison with hypertensive participants (baseline systolic ≥140 mmHg).


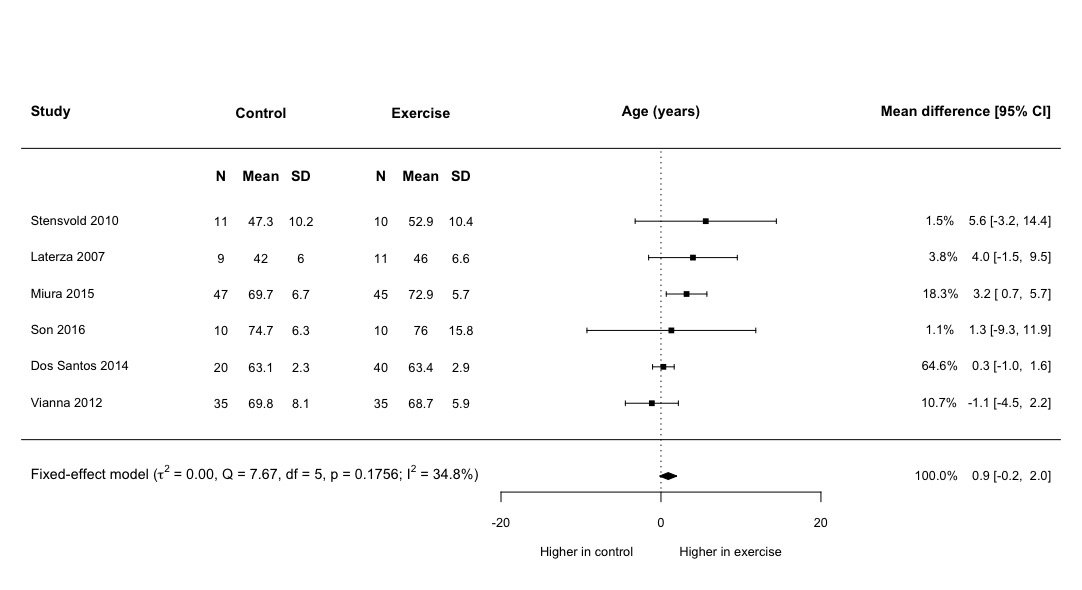


**Supplementary Figure 6.** Forest plot for age for the combined vs control comparison with hypertensive participants (baseline systolic ≥140 mmHg).
